# Consciousness and inward electromagnetic field interactions

**DOI:** 10.3389/fnhum.2022.1032339

**Published:** 2022-11-17

**Authors:** M. Bruce MacIver

**Affiliations:** Stanford University School of Medicine, Palo Alto, CA, United States

**Keywords:** mind, electrodynamic, chaos, ephaptic, quantum fields

## Abstract

Electromagnetic field (EMF) theories of mind/brain integration have been proposed to explain brain function for over seventy years. Interest in this theory continues to this day because it explains mind-brain integration and it offers a simple solution to the “binding problem” of our unified conscious experience. Thus, it addresses at least in part the “hard problem” of consciousness. EMFs are easily measured and many corelates have been noted for field activity; associated with loss and recovery of consciousness, sensory perceptions, and behavior. Unfortunately, the theory was challenged early on by experiments that were thought to have ruled out a role of EMFs in brain activity, and the field of neuroscience has since marginalized EMF theories. Here I explain why early evidence against EMFs contributing to consciousness was misinterpreted and offer an alternative view to help direct future research.

## Introduction

**FIGURE 1:**
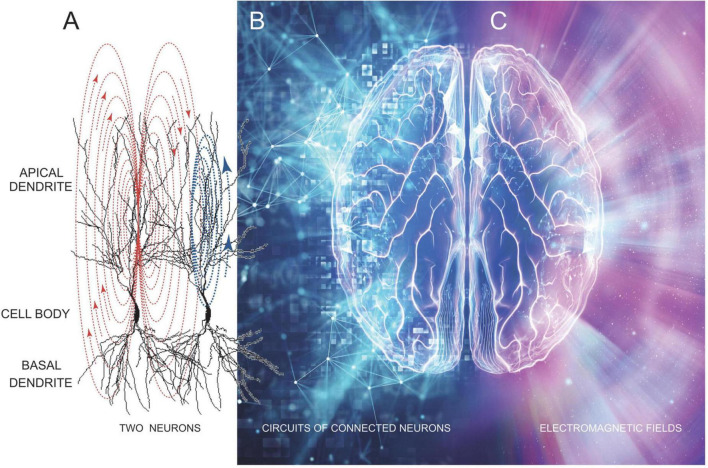
A stylistic representation showing how brain neural circuitry **(A,B)** underlies the generation of EMFs **(C)** that unifies brain electrical activity into a conscious whole–that is our mind. Image was modified from freepik.com.

Electromagnetic field (EMF) theories of mind/brain integration posit that current flow across neuronal membranes generates an electromagnetic field which, in turn, permits computation and integration of information, that produces a conscious mind (Pockett, 2014; McFadden, 2020). Thus, consciousness arises from a dynamic electromagnetic field that reflects synaptic and discharge currents of neurons throughout the brain (Köhler and Held, 1949; Jones, 2013). The prevailing idea is that the EMF forms an aura-like three-dimensional energy cloud emanating from our brains, and extending beyond our skulls, where it can be recorded as EEG and/or MEG signals that exhibit complex patterns (Figure 1). I present a new way to visualize these complex patterns, using non-linear dynamic analyses of EEG recordings. 3-D plots of phase information derived from EEG signals nicely track levels of consciousness in humans and animals (MacIver and Bland, 2014; Eagleman and MacIver, 2021). These findings support EMF theories of consciousness, but provide only a crude measure of the complexity and integrating power of EMFs because they only measure the least powerful and most diffuse part of our conscious energy field.

## Hypothesis

The standard view of brain/mind integration is illustrated in Figure 1, showing how neuronal circuit electrical activity in the brain produces a cloud of energy which radiates as our brain’s EMF. The idea being that synaptic and discharge currents in neurons (Figure 1A), especially large numbers of connected and synchronously and often rhythmically active neurons (Figure 1B) produce an EMF “cloud” of energy that changes moment by moment as underlying brain electrical activity changes. There is no doubt that this energy cloud exists since it is easily measured using both electrical and magnetic detectors (EEG and MEG) (Dyba et al., 2021; Keppler, 2021; Young et al., 2021, 2022; Hales and Ericson, 2022; Kitchener and Hales, 2022). From these measures we know EMFs radiate in 3 dimensions at varying powers and frequencies, represented in Figure 1C as differing colors on the right side of the image.

Electromagnetic fields are produced (generated) by neurons that are connected by chemical and/or electrical synapses, as well as *via* ephaptic connections. In Figure 1A, an ephaptic connection between two nearby neurons is shown by the synaptic current flow in apical dendrites of the first neuron (red arrows) inducing a depolarizing current flow in an adjacent neuron (blue arrows). Electrical synapses connect dendrites of adjacent neurons *via* gap junctions that are essential for the generation of EEG rhythms (Gołebiewski et al., 2006; Bocian et al., 2011). Larger groups of neurons are connected into circuits, mostly *via* chemical synapses, which are thought to underlie memory engrams and brain computational units (Figure 1B). When these circuits are active they produce synchronized synaptic and discharge activity across wide regions of the brain. This synchronized neuronal activity summates to generate the EMFs we record as EEG and MEG signals (Figure 1C). We know these fields extend for relatively long distances because they can be measured through at least the 7 mm of the human skull and scalp.

The strengths, supporting evidence, and utility of an EMF model have been well reviewed, and a number of proposed weaknesses were refuted by McFadden (2020) as well as in a scholarpedia article (Pockett, 2022). However, the earliest refutations of EMF theories have not been well addressed (Lashley et al., 1951; Sperry et al., 1955). A question remains, why do not EMF shields or perturbations of the EMF affect mental processes?

Placing gold leaf shields or other conductive materials (i.e., electrode arrays) on the brain’s surface, to short circuit (shunt) electric current flow, should disrupt or deteriorate an EMF such that an effect on mental processing would be apparent. Yet experiments testing this have failed to show altered mental processes (Lashley et al., 1951; Sperry et al., 1955; Endemann et al., 2022). Why not?

**FIGURE 2:**
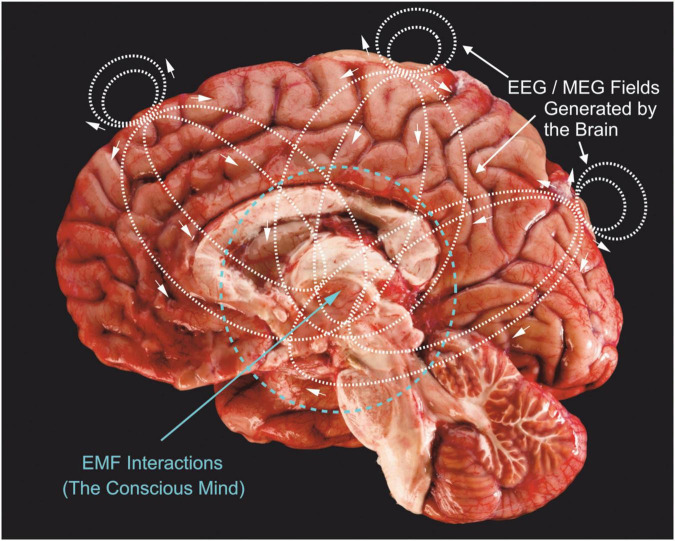
Three current sources (e.g., synaptic ion channels) located in upper cortical layers of frontal, midline and occipital areas are shown, together with the charge paths they would produce (dotted lines with small arrows). Synaptic currents are usually carried by positive charges (Na^+^ and Ca^++^) that enter cells across dendritic membranes, so current lines show negative charge movement. These charge paths flow bidirectionally to produce fields above the cortex; these are what we measure with EEG surface electrodes. There are also charge paths directed inward, toward the thalamus and brainstem regions. Inward directed charge pathways would extend further because they are propagating through an electrolytic media that is not impeded by the dura and skull. I propose that these current paths would be concentrated toward the center of brains and generate much stronger EMFs compared to outward directed fields. Brain image modified from ProProfs.com.

Figure 2 shows the electric current lines generated by just 3 synaptic current sink/sources located in three neocortical regions: frontal, midline and occipital areas. Of course, this is a very simplistic view of actual field generators which are active over wide ranging cortical and subcortical neuronal generators in an ever-changing pattern of complex current paths. The associated magnetic fields are not shown, but would be perpendicular to these current lines. Electrical and magnetic paths radiate in three dimensions, not just in the two dimensions shown in Figure 2.

Since synaptic current will take the path of least resistance through interstitial fluid and membranes of the brain, they will extend further into the brain, rather than outward, due to the increased resistance of our skull tissue. Measurements comparing deep electrode responses to surface electrode recordings consistently demonstrate similar or higher signal amplitudes (Hashimoto et al., 1981; Mishra et al., 2021). If the mind is “located” in these centralized overlapping EMFs, then it provides a stronger possibility of unification, “binding” very divergent brain activities into a central whole (Kitchener and Hales, 2022). We can also see (Figure 2) that outward fields would play only a small role in modifying neuronal activity through ephaptic influences on neurons. This is critical because we know that EMFs influence the discharge of neurons and this closes a loop for mind-brain duality by linking EMF energy back to controlling neuronal discharge (McFadden, 2013). The increased and focused density of inward directed EMFs would provide stronger ephaptic control of neurons, especially those in the brain’s central regions. Placing shields or introducing EMFs from external sources outside of the skull would hardly alter inward directed energies and would, hence, not appreciably alter our brain, mind or consciousness; this is what has been observed experimentally (Lashley et al., 1951; Sperry et al., 1955). Similarly, implanted deep brain stimulating electrodes appear to produce too localized a perturbation to alter the mind’s EMF, although effects on cognition do occur in some patients (Agashe et al., 2022; Chang et al., 2022). It has long been known that high strength magnetic fields of MRI scanners effect both human and animal subjects, producing dizziness, altered behavioral responses and cognitive impairment (Antunes et al., 2012; Tkáč et al., 2021). Weaker EMFs produced by cell phones, radios and headphones do not appreciably alter mental activity, although long-term exposure to these weak fields may disrupt some brain functions (Bodewein et al., 2022; Schüz et al., 2022). Transcranial magnetic stimulation using strong magnetic pulses are well known to stimulate brain neurons, as does ultrasonic stimulation (Sarasso et al., 2015; Sanguinetti et al., 2020). EMF perturbations on the surface of the brain or outside it or very close to the surface do not affect mental processes, whereas EM perturbations that penetrate deeply into the brain do affect mental process.

**FIGURE 3:**
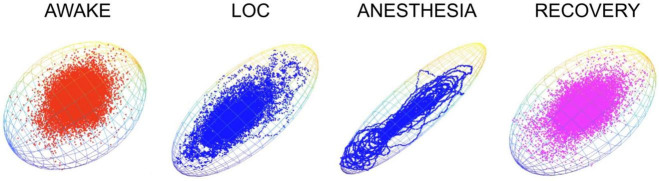
Chaotic attractors provide a sensitive measure of levels of consciousness in subjects exposed to an anesthetic. EEG signals were recorded from frontal cortex and processed as previously described (Eagleman et al., 2018b). Attractor plots produce spherical clouds in awake subjects. Loss of consciousness (LOC) is associated with a flattening of the attractor cloud. A further flattening is seen at surgical planes of anesthesia, with a return to a more spherical shape upon recovery, following removal of the anesthetic. Grids indicate best fit boundaries for each 3-D matrix. Unpublished data from Eagleman and MacIver.

Non-linear (chaos) analysis of EEG signals provide a measure of our brain’s electric field, and also provide a sensitive measure of human consciousness (Watt and Hameroff, 1988; Walling and Hicks, 2006). We have recently explored a new way to visualize the chaotic complexity of brain activity from EEG signals; as chaotic attractor 3-D clouds which capture phase and complexity information and track the level or degree of consciousness in subjects slowly anesthetized, and then allowed to recover (Figure 3). When subjects are awake and conscious, 3-D clouds are largely spherical, reflecting many degrees of freedom and complex brain activity. As loss of consciousness (LOC) is produced, either through sleep, or in this case following anesthetic exposure, 3-D clouds begin to collapse into ellipsoid shapes. Deepening anesthesia, beyond loss of consciousness, results in further flattening of the clouds until cigar-like clouds are seen at deep surgical planes of anesthesia. Flattened clouds readily return to more spherical shapes upon recovery and awakening (Eagleman et al., 2019).

What can these attractor clouds tell us about the EMF that is generated by our brains? They certainly provide a way to visualize the electric field component of the EMF since they are derived from EEG (i.e., electrical) signals, but they contain no magnetic information. Magnetic (MEG) signals have yet to be analyzed with this method. Attractor clouds are thought to reflect the complexity and information content of signals, with higher information being associated with more spherical plots. A more spherical plot indicates higher degrees of freedom in EEG signals, allowing the attractor to explore more regions of the complexity landscape. The anesthetic-induced collapse of the attractor certainly fits with an anesthetic-induced collapse of information integration that occurs at loss of consciousness (Oizumi et al., 2014; Sarasso et al., 2015; Tononi et al., 2016; Eagleman and MacIver, 2018, 2021; Eagleman et al., 2018a,b, 2019; Ward and Guevara, 2022). A more sophisticated approach would measure both electric and magnetic fields, together with photonic energies (Salari et al., 2021), and combine these into multi-dimensional attractors. Even better would be approaches which allow us to record EMFs from deeper regions of the brain, like the thalamus, midbrain and brainstem, together with cortical level signals. This would provide an enriched view of brain function and the distribution of EMFs throughout our higher nervous system regions.

## Discussion

Looking inward at EMF energy clouds, as opposed to the outward view most of us have envisioned, can readily account for why external fields and shielding do not alter mental processes. This view also supports the idea that EMFs focused into the brain would provide stronger ephaptic connections to the brain’s neural circuits. Providing a stronger coupling between energies of the brain and the mind, from quantum energies of photons and particles, to atoms, molecules, microtubules, synapses and circuits of cells; to energy fields and conscious thought; and back again (Hameroff and Penrose, 2014; McFadden, 2020; Hameroff, 2022).

## Data availability statement

The original contributions presented in this study are included in the article, further inquiries can be directed to the corresponding author.

## Author contributions

MM researched and envisioned this manuscript in its entirety and wrote and revised the manuscript as well as compiled the illustrations.
